# Integrating Genetic and Transcriptomic Data to Reveal Pathogenesis and Prognostic Markers of Pancreatic Adenocarcinoma

**DOI:** 10.3389/fgene.2021.747270

**Published:** 2021-09-09

**Authors:** Kaisong Bai, Tong Zhao, Yilong Li, Xinjian Li, Zhantian Zhang, Zuchao Du, Zimin Wang, Yan Xu, Bei Sun, Xuewei Bai

**Affiliations:** ^1^Department of General Surgery, The First Affiliated Hospital of Harbin Medical University, Harbin, China; ^2^Key Laboratory of Hepatosplenic Surgery, Ministry of Education, Harbin, China; ^3^School of Life Sciences and Technology, Harbin Institute of Technology, Harbin, China; ^4^Department of Pancreatic and Biliary Surgery, The First Affiliated Hospital of Harbin Medical University, Harbin, China

**Keywords:** pancreatic cancer, somatic mutation, genomic variation, prognostic marker, complex disease

## Abstract

Pancreatic adenocarcinoma (PAAD) is one of the deadliest malignancies and mortality for PAAD have remained increasing under the conditions of substantial improvements in mortality for other major cancers. Although multiple of studies exists on PAAD, few studies have dissected the oncogenic mechanisms of PAAD based on genomic variation. In this study, we integrated somatic mutation data and gene expression profiles obtained by high-throughput sequencing to characterize the pathogenesis of PAAD. The mutation profile containing 182 samples with 25,470 somatic mutations was obtained from The Cancer Genome Atlas (TCGA). The mutation landscape was generated and somatic mutations in PAAD were found to have preference for mutation location. The combination of mutation matrix and gene expression profiles identified 31 driver genes that were closely associated with tumor cell invasion and apoptosis. Co-expression networks were constructed based on 461 genes significantly associated with driver genes and the hub gene FAM133A in the network was identified to be associated with tumor metastasis. Further, the cascade relationship of somatic mutation-Long non-coding RNA (lncRNA)-microRNA (miRNA) was constructed to reveal a new mechanism for the involvement of mutations in post-transcriptional regulation. We have also identified prognostic markers that are significantly associated with overall survival (OS) of PAAD patients and constructed a risk score model to identify patients’ survival risk. In summary, our study revealed the pathogenic mechanisms and prognostic markers of PAAD providing theoretical support for the development of precision medicine.

## Introduction

Pancreatic adenocarcinoma (PAAD) remains one of the deadliest cancer types and has become the leading cause of cancer-related mortality in the United States (Rahib et al., 2014; Ilic and Ilic, 2016). The incidence and mortality rates of PAAD vary widely worldwide and are highest in developed countries (McGuigan et al., 2018). Although studies have shown that smoking, obesity, hereditary diabetes and irregular diet are risk factors for the development of pancreatic cancer, the pathogenesis was still poorly understood. Several of treatments exist that can improve the prognosis of PAAD patients. For example, nab-paclitaxel plus gemcitabine (Von Hoff et al., 2013) and FOLFIRINOX vs. gemcitabine (Conroy et al., 2011). Although these treatments have improved the survival of some patients, the 5-year survival rate of PAAD still remains severe at 8% (Siegel et al., 2017). Therefore, it is necessary to deeply discover the carcinogenic mechanism and possible therapeutic targets of PAAD.

Genomic variation refers to differences in the structure and composition of DNA between individuals or between populations. With the development of high-throughput sequencing, multiple sources of disease-related genomic variation have been identified such as copy number variation and somatic mutations. Large-scale cancer genome sequencing consortia, such as The Cancer Genome Atlas (TCGA) (Tomczak et al., 2015) and ICGC (International Cancer Genome et al., 2010), have provided somatic mutation data from numerous of tumor patients. The role of somatic mutations in the development of specific cancer phenotypes is the main purpose of cancer genomics studies (Vogelstein et al., 2013). Somatic mutations have significant tumor heterogeneity, and each individual has different sets of mutations across many genes. Therefore, exploring the mutation-driven regulation of gene expression can better serve the purpose of precision medicine.

Work from the past decade has given us a whole new perspective on non-coding RNAs. For example, Long non-coding RNA (lncRNA) have been demonstrated to play an important role in chromatin reprogramming, transcription, post-transcriptional modifications and signal transduction (Anastasiadou et al., 2018; Wang et al., 2021). LncRNA could act as a miRNA sponge to participate in competitive endogenous RNA (ceRNA) regulation determined by microRNA (miRNA) response elements (MREs) (Salmena et al., 2011), which is an important way for it to regulate gene expression post-transcriptionally. Somatic mutations in the MRE region of the lncRNA may weaken, enhance or prevent binding to the pro-miRNA, which may cause some imbalance in the ceRNA regulatory network and even alter the expression of related target genes in the regulatory pathway (Thomas et al., 2011; Thomson and Dinger, 2016).

Here, we have collected mutation data, clinical information and transcript expression profile of PAAD from TCGA to conduct a systematic investigation concerning mutation features, pathogenesis and prognostic markers.

## Materials and Methods

### Data Collection

The somatic mutation profiles (182 samples), clinical information (222 samples), and RNA-seq profiles (178 tumor and 4 paracancer samples) of PAAD were collected from TCGA (Tomczak et al., 2015).^1^ We collected hallmark gene sets from the molecular feature database (MSigDB v7.4 Liberzon et al., 2015)^2^ for enrichment analysis of carcinogenic functions. The human genome annotation data of GRCh38 v29 version including the position and sequence information of lncRNA was collected from GENCODE (Frankish et al., 2019)^3^. The sequence information of 2654 miRNA was obtained from miRbase v22 (Kozomara et al., 2019)^4^ database. Further, we downloaded the experimentally validated miRNA-target gene regulatory relationships from miRTarBase v8.0 (Chou et al., 2018)^5^ to reconstruct ceRNA regulatory relationships.

### Statistical Analysis of Somatic Mutations

The R package maftools (version 2.8.0) (Mayakonda et al., 2018) was used for the statistical and visualization of mutation location, mutation form, mutation frequency and other information. The package enables efficient aggregation, analysis, annotation and visualization of MAF files from TCGA sources or any in-house study. We also used the visualization results of maftools to reveal new discoveries of PAAD.

### Driver Gene Identification

We first counted the number of mutations in each gene across samples to generate a mutation matrix. Combined with the gene expression profile of PAAD from TCGA, we retained genes that were mutated in at least two samples. Further, the difference in expression of each gene between mutated and unmutated samples was measured by Student’s *t*-test and fold change. We set the cutoff for *p*-value and fold change to 0.05 and 1.5, respectively (He et al., 2021). We define genes that are differentially expressed between mutated and unmutated samples as mutation driver genes.

### Construction of Gene Co-expression Networks

For the driver genes affected by mutations, we separately calculated other genes co-expressed with each driver gene, which may interact with each other and play a role in the occurrence and development of PAAD. Pearson’s (Bishara and Hittner, 2012) correlation algorithm was used to calculate the correlation between the expression of two genes, which was performed by cor.test function of R. We defined gene pairs with *p-*value < 0.01 and correlation coefficient | R| > 0.5 as those with significantly related expression. For all co-expressed genes, cytoscape (v3.7.0)^6^ (Shannon et al., 2003) was used to plot the co-expression network. Further, NetworkAnalyzer was used to calculate the topological properties of the network and to mark the size of the nodes according to their degree.

### Identification of Putative Mutation-miRNA-lncRNA Regulation Units

Somatic mutations occurring in lncRNA may affect the affinity of the original lncRNA and miRNA binding (Wang P. et al., 2020; Zhang et al., 2021). Based on the lncRNA annotation information collected from GENCODE (v29, GRCH38), we relocated the mutations that occurred in the lncRNA. Considering the requirements of miRNA target prediction tools for predicted sequences, we extracted sequences of 21 approximately nucleotides (nt) upstream and 7 nt downstream of the lncRNA somatic mutation site, which will be used to construct mutation and wild sequences. TargetScan (v.6.0)^7^ and miRanda (v2010),^8^ which are two miRNA target prediction tools, were used to predict the possible combination of miRNA and mutant/wild sequence. We also set stringent thresholds of score > 160 and energy < −20 for miRanda (Betel et al., 2008) and context score < −0.4 for TargetScan (Friedman et al., 2009), and miRNA-targets that satisfy this threshold are considered to be reliable. We define mutations that affect the affinity of miRNA binding to wild sequences as putative mutations, and the lncRNA in which the putative mutation was located as ceL. Further, the altered binding affinity of miRNA and mutation/wild sequence was divided into four states including gain, up, loss, and down. For these ceRNAs perturbed by somatic mutations, we constructed putative mutation-miRNA-lncRNA (ceL) units.

Next, altered binding affinity of the original lncRNA and miRNA may affect the expression of other downstream mRNAs regulated by this miRNA (Wang et al., 2015; Wang P. et al., 2019; Zhang et al., 2021). We collected miRNA-target mRNA regulatory relationships from the miRTarBase database that were validated by experiments including the luciferase reporter assay, PCR, and western blotting to build the somatic mutation-lncRNA-miRNA-mRNA (ceRNA dysregulation) network.

### Functional Enrichment Analysis

For those mutated genes, we sorted the genes with a weight of −log10(*p-*value). The sorted genes and hallmark gene set were used for gene set enrichment analysis (GSEA) (Subramanian et al., 2005). Similarly, for those genes co-expressed with the mutation driver genes, we ordered the co-expressed genes for each driver gene using the correlation coefficient as a weight, which was also used for GSEA. The clusterprofiler (v3.18.0) (Yu et al., 2012) R package was used to perform gene ontology (GO) functional enrichment and kyoto encyclopedia of genes and genomes (KEGG) pathway analysis on these mRNA. We set *p-*value < 0.05 to screen for significantly enriched functions and pathways.

### Constructing Survival Prediction Model

We integrated significantly differentially expressed mutant genes (*p*-value < 0.05 only) and other protein-coding genes perturbed by putative mutations in these genes through the ceRNA mechanism. First, we used univariate COX regression to screen for genes significantly associated with overall survival (OS) in PAAD patients (the cutoff of *p-*value was 0.05). Considering that univariate cox regression was not sufficiently rigorous, lasso regression (Alhamzawi and Ali, 2018) was used to further screen for prognosis-related genes. Next, we randomly selected 70% of all samples as the training set and the remaining as the testing set. The train set were used to construct a multivariate COX regression model (Fisher and Lin, 1999). The Hazard Ratio hypothesis test was also used in the construction of the regression model. We retained the genes passing the Hazard Ratio hypothesis test to establish survival risk prediction model and nomogram to predict the OS of PAAD. The reliability of this risk prediction model was depicted by the receiver working characteristic curve (ROC), and the area under curve (AUC) also was calculated. The train set and test set was, respectively, divided into high-risk and low-risk groups based on the median risk score calculated by risk score model, and Kaplan-Meier (KM) survival analysis was used to measure the difference in OS between these two groups and bilateral logarithmic rank test was used.

### Statistical Analysis

All statistical analyses and graph generation were performed in R (version 4.0.2). The R package resources were obtained from http://www.bioconductor.org/ and https://cran.rstudio.com/bin/windows/Rtools/.

## Results

### The Landscape of Pancreatic Adenocarcinoma Somatic Mutations

**FIGURE 1:**
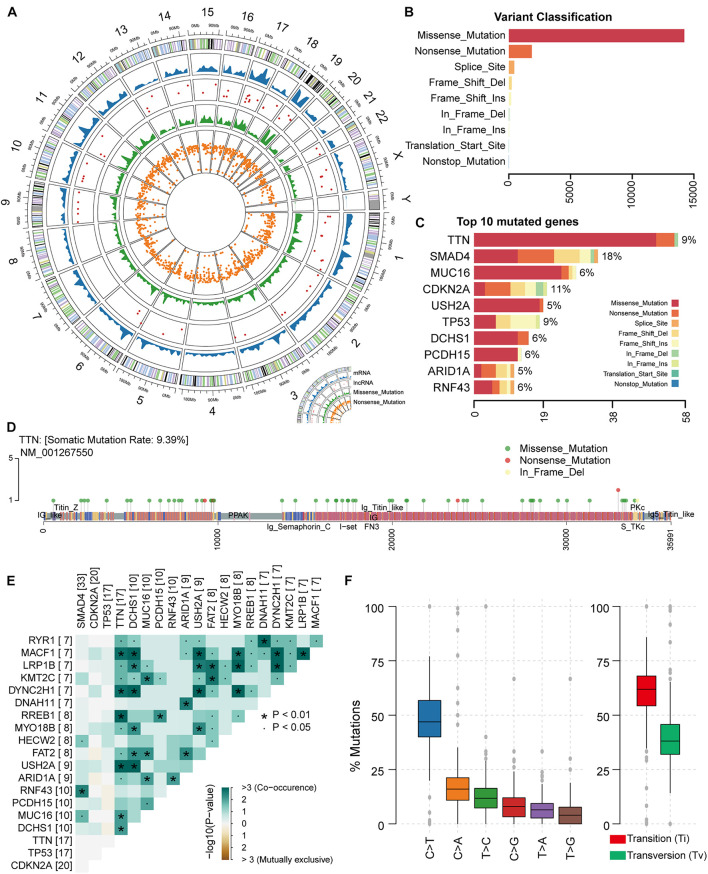
Genomic overview of somatic mutations in PAAD. **(A)** The global view of genomic variations location. **(B)** Somatic mutations were classified into nine clusters according to function and location. The bar plot shows the number of mutations in each cluster. **(C)** The bar plot illustrates the proportion of each cluster of mutation in the top 10 genes in terms of number of mutations. The proportion of samples to which the mutation on each gene belongs was also calculated. **(D)** The location and type of mutations occurring on gene TTN were shown by the lollipop chart. **(E)** The mutation correlation between the top 20 high-frequency mutated genes. **(F)** The frequency of base substitutions (transitions and transversions) in PAAD.

In this study, it is necessary to perform an overall statistical analysis of the somatic mutations in PAAD. First, we evaluated samples in the TCGA database collection for which somatic mutation data were available. The result contained 182 samples with 25,470 somatic mutations. We counted the distribution of somatic mutations on the genome including chromosomal location and transcript type. We found that somatic mutations were significantly enriched on chromosomes 17 and 19 (Figure 1A), suggesting the preference of PAAD somatic mutation in the mutation position. Compared with transcripts (mRNA) of protein-coding genes, several somatic mutations occur in lncRNA (Figure 1A). Although relatively few mutations occur in the non-coding region, studies have confirmed that mutations within the non-coding genome are a major determinant of human disease (Maurano et al., 2012). Somatic mutations, including missense and nonsense mutations, account for the largest proportion of all somatic mutations, with missense mutations predominating (Figures 1A,B). We also found mutations occurring at the transcription start site in only four samples (Figure 1B). All these suggest that PAAD patients are more likely to have mutations that alter protein function to disrupt normal physiological mechanisms. Further, we counted the frequency of mutations in each gene and the number of samples with mutations in that gene, and the top mutated genes were illustrated (Figure 1C and Supplementary Figure 1A). We found that different genes have different preferences in the type of mutation. For example, *TTN*, the gene considered to be most frequently mutated in the pan-cancer cohort (Oh et al., 2020), tended to have missense mutations in PAAD, whereas the *TP53* gene had a high proportion of indel mutations. Studies have shown that the impact of mutations on the prognosis of patients is related to the type and background of the tumor (Hainaut and Pfeifer, 2016). As a mutated gene commonly occurring in PAAD patients, *TTN* has multiple non-sense mutation hot spots (Figure 1D), which will have a significant impact on the function and structure of its encoded protein. We found no significant exclusivity between high-frequency mutated genes in the PAAD samples, and a general correlation between the *TNN* gene and other high-frequency mutated genes (Figure 1E), revealing a mutational feature of pancreatic cancer that the coordinated mutation of multiple genes affects the normal physiological mechanism. We found that nearly half of the point mutations (base substitution) in PAAD patients are C > T substitutions (Figure 1F and Supplementary Figure 1B). Transitions, one of the two types of DNA base conversion, have a high proportion of overall PAAD point mutations, which are capable of being retained by evolution. However, transversions as another type of DNA base conversion account for nearly 30% of overall point mutations, and these mutations may be key factors in the deterioration of pancreatic tissue. Taken together, all these revealed the mutational features of PAAD.

### Driver Genes Boost Tumor Invasion

**FIGURE 2:**
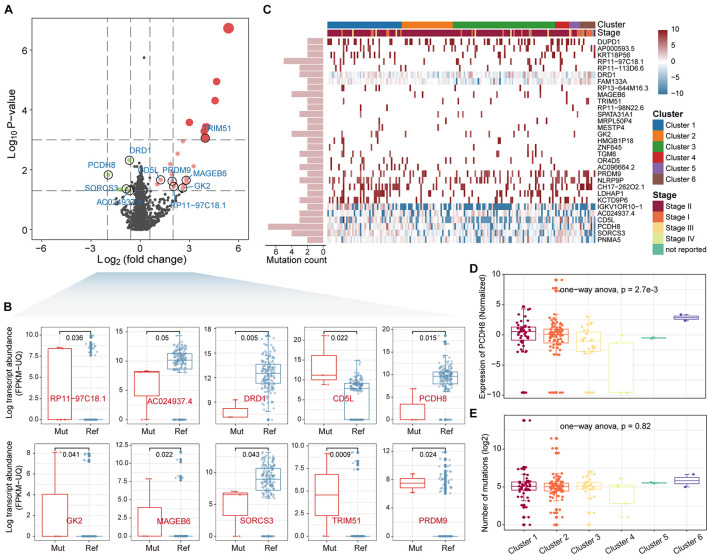
Identification of driver genes. **(A)** The differential expression analysis comparing mutated and unmutated patients. Differentially expressed genes were shown as red or green dots. **(B)** Expression level of the top 10 driver genes between mutated and unmutated samples was shown by boxplot. The rank sum test was used to check the significance. **(C)** Distribution of driver gene expression in the samples. Column labels indicate the cluster and stage to which the sample belongs. Row labels indicate the number of mutations in each driver gene. **(D,E)** Expression and mutation levels of the driver gene PCDH8 between the 6 clusters of samples were shown by boxplot.

Somatic mutations could indirectly affect biological traits by regulating gene expression. It is thus intriguing to explore genes whose expression changes affected by mutations. We integrated the mutation and gene expression profiles of PAAD, with 173 samples having both mutation and gene expression data. A total of 4,517 genes that were mutated in at least two samples were collected to construct the mutation matrix. By comparing the differential expression of each gene between mutant and non-mutant samples, we identified a total of 31 driver genes that were significantly differentially up/down regulated [*p*-value < 0.05, | log2(fold change) | > log2(1.5)] (Figure 2A). We next sorted the genes by fold change. The top 10 driver genes were *RP11-97C18.1 (ENSG00000225191)*, *AC024937.4 (ENSG00000231464), DRD1, CD5L, PCDH8, GK2, MAGEB6, SORCS3, TRIM51*, and *PRDM9* (Figure 2B). The top driver gene *RP11-97C18.1* is a pseudogene of Adaptor-Related Protein Complex 2, Beta 1 Subunit (*AP2B1*), which is an essential adaptor of the clathrin-mediated endocytosis pathway (Diling et al., 2019; Wang G. et al., 2020). The driver gene *AC024937.4* is also a pseudogene of ADP-ribosylation factor-like 8B (*ARL8B*), which is involved in cellular endocytosis, autophagy and the movement of phagocytic vesicles on microtubule tracks to fuse with lysosomes (Marwaha et al., 2017). All these suggest non-coding genes are essential in the development and progression of PAAD. Further, consensus clustering tools were used to cluster PAAD samples based on driver gene expression profiles. These samples were divided into six clusters (Supplementary Figure 2). We found that *PCDH8*, which acts as a tumor-suppressor gene in multiple types of cancer and inhibits tumor cell proliferation, invasion and migration (Yu et al., 2020), was downregulated in clusters 3 and 4 (Figures 2C,D), suggesting that tumor cells may be more aggressive in the two clusters with lower *PDCH8* expression. Patients in stage I were mainly concentrated in clusters 5 and 6 (Figure 2C). It is intriguing that there is no significant difference in the number of sample mutations in each cluster (Figure 2E), revealing that differences in gene expression of samples among clusters are not simply determined by the number of mutations. Taken together, all these suggest that driver genes affected by mutations play an essential role in the proliferation and invasion of PAAD.

### Interaction of Essential Factors With Driver Genes Regulates Oncogenic Pathways

**FIGURE 3:**
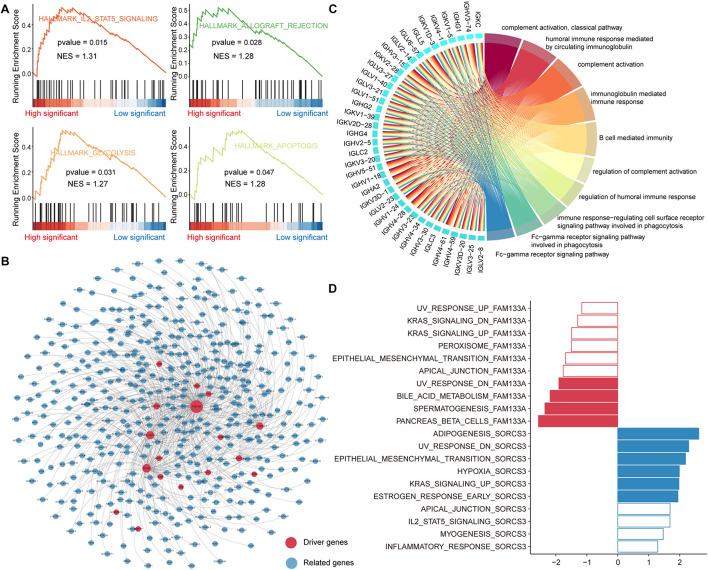
Functional enrichment analysis of driving related genes. **(A)** The GSEA analysis of mutant genes, which explain somatic mutations in the genome of PAAD patients. **(B)** Co-expression network of driver genes and their significantly related genes. The size of the node is related to the degree. **(C)** The top 10 GO items that are enriched by genes significantly related to driver genes. This network shows the interaction between genes and GO items. **(D)** Top 10 hallmark pathways enriched in genes related to driver gene FAM133A or SORCS3. significantly enriched pathways with GSEA *p*-values < 0.05 are highlighted in red (FAM133A) or blue (SORCS3).

For those genes that were mutated, they may play an essential role in the proliferation and invasion of tumors. In order to explore the role of these genes in carcinogenic pathways, we performed GSEA to identify hallmark pathways enriched in mutant genes explaining somatic mutations in the genome of PAAD patients (see section “Materials and Methods”). We found that IL2-STAT5 signaling, glycolysis, apoptosis and allograft rejection pathways are significantly enriched in genes whose expression is affected by somatic mutations (Figure 3A). Studies have shown that interleukin-2 (IL-2) and the downstream transcription factor STAT5 are essential for maintaining regulatory T (Treg) cell homeostasis and function (Cheng et al., 2018), suggesting that the immune microenvironment in tumor tissue of PAAD patients affected by somatic mutations may be disrupted. The altered glycolytic machinery in PAAD was designed to adapt to the tumor microenvironment, which is consistent with previous studies showing that cancer cells are preferentially dependent on glycolysis (Ganapathy-Kanniappan and Geschwind, 2013). The allograft rejection pathway affected by mutations may become the key point of PAAD immunotherapy (Land et al., 2016).

Global reprogramming of the transcriptome occurs in order to support tumorigenesis and progression. In addition to the direct effect of mutations on gene expression, there are other regulatory mechanisms such as transcriptional regulation, ceRNA mechanisms, epigenetic. Genes co-expressed with driver genes may have a potential role in tumor development. We performed the Pearson correlation algorithm to identify genes that may be influenced by other regulatory mechanisms co-expressed with driver genes. We identified 495 genes (491 positive and 4 negative) significantly associated with 19 driver genes (*p*-value < 0.01, | R| > 0.5). These significantly related genes were used to construct gene co-expression networks using cytoscape (Figure 3B). We also counted the topological properties of the network using the NetworkAnalyzer tool and found that the gene *FAM133A* had the top degree (Supplementary Table 1). *FAM133A* has been confirmed in previous studies to be related to the invasion and metastasis of glioma (Huang et al., 2018). Next, we performed a functional enrichment analysis of all genes in the co-expression network using the R package clusterprofiler. We found that these genes were significantly enriched in immune-related functions and apoptotic pathways, such as complement activation, immunoglobulin mediated immune response, B cell mediated immunity, and apoptosis—multiple species (Figure 3C and Supplementary Figure 3). For the 19 driver genes identified as having co-expressed genes, we used GSEA to analyze the functional features of the driver genes. Hallmark gene sets and genes ordered by correlation coefficients were available for GSEA. We found that the oncogenic pathway was significantly enriched only in genes co-expressed with the driver genes *FAM133A* and *SORCS3*, suggesting that most driver genes are required to synergistically regulate oncogenic mechanisms. In contrast to the driver gene *FAM133A*, the driver gene *SORCS3*, in combination with its co-expressed genes, plays an important role in tumor metastasis, hypoxia and apoptosis (Figure 3D). Taken together, all these indicate that the synergistic interaction network of multiple driver genes may contribute to the complex pathogenesis of PAAD.

### LncRNA Mutations-ceRNA Indicates Novel Mechanisms of Mutation Regulation

**FIGURE 4:**
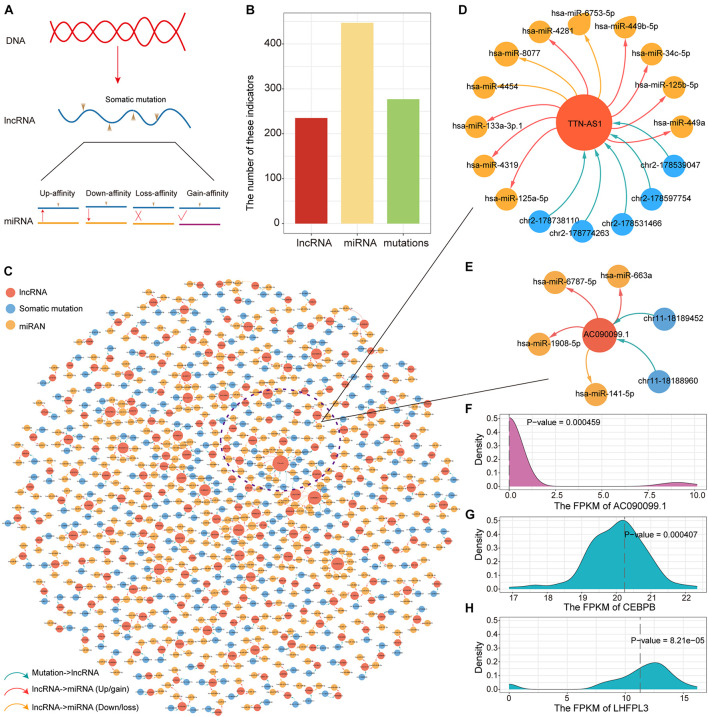
Construction of the ceRNA dysregulated network. **(A)** Mutations in lncRNA affect binding intimacy with miRNA. **(B)** The number of lncRNA, miRNA and somatic mutations in the ceRNA dysregulated network. **(C)** The ceRNA dysregulated network constructed by lncRNA, miRNA, and somatic mutations. The red, blue and yellow nodes represent lncRNA, somatic mutation and miRNA, respectively. Up-regulated or newly gain affinity between miRNA and lncRNA used red line. Down-regulated or loss affinity between miRNA and lncRNA used yellow line. **(D)** The ceRNA dysregulated sub-network of lncRNA TTN-AS1. **(E)** The ceRNA dysregulated sub-network of lncRNA AC090099.1. **(F–H)** The distribution of the expression for lncRNA AC090099.1, CEBPB, and LHFPL3 was shown by density curve. The expression value of these genes in the mutant sample were marked with a red line. One-sample *t*-test was used to calculate statistical significance.

LncRNA have been confirmed that genes are essential in pre- and post-transcriptional regulation. The lncRNA with (miRNA) response element (MRE) can be used as a miRNA sponge to participate in the ceRNA regulatory mechanism. To explore the impact of somatic mutations occurring on lncRNA MREs on ceRNA regulatory mechanisms, we constructed mutant/wild sequences to identify mutations that alter the affinity of lncRNA-miRNA binding. Based on lncRNA annotation data collected from GENCODE, we identified 497 somatic mutations occurring on lncRNA compared to 24,604 somatic mutations occurring on the genome. Affected by mutations, lncRNA may enhance, reduce and lose their binding affinity to existing miRNAs, or even gain binding affinity to new miRNAs (Figure 4A). Next, we examined the influence of lncRNA mutations on miRNA binding sites according to the TargetScan and miRanda. In total, we identified 277 somatic mutations for PAAD in 235 putative miRNA target genes (putative lncRNAs). These mutation sites showed different binding affinities to 447 miRNAs between the mutation and wild sequences (Figure 4B). All these constituted 552 mutation-miRNA-lncRNA regulation units. We further constructed ceRNA dysregulation networks based on the identification of mutation-miRNA-lncRNA regulation units (Figure 4C). We found that TTN-AS1 has top degree in the ceRNA dysregulation networks and that five somatic mutations occurring on it affect the affinity of binding to 11 miRNAs (8 Up/gain and 3 Down/loss, Figure 4D). Combining 31 driver genes, we found that only driver lncRNA *AC090099.1 (ENSG00000255470)* has mutations involved in ceRNA regulation imbalance, which suggesting that the mechanisms underlying changes in driver gene expression are complex. We found two mutations in *AC090099.1* that affected binding affinity to four miRNAs (3 Up/gain and 1 Down/loss, Figure 4E). In order to verify our prediction results at the transcriptome level, we performed one-sample *t*-test to identify the difference between the gene expression level of the non-mutated sample and the mutant sample. We found significant differences in the expression of *AC090099.1* and the target gene *CEBPB* and *LHFPL3* regulated by miRNA *hsa-miR-663a* between mutated and unmutated samples (Figures 4F–H). Taken together, all these results suggest that ceRNA dysregulation due to lncRNA mutations is an essential factor in variations of target gene expression.

### Identifying Prognostic Markers for PAAD

**FIGURE 5:**
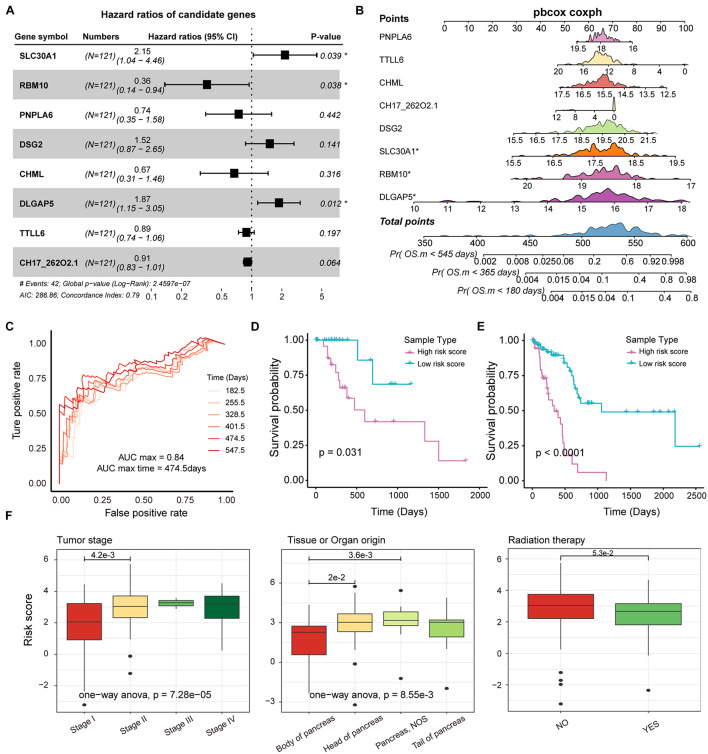
Survival analysis of potential markers in PAAD. **(A)** Forest plots for multivariate Cox risk regression models. **(B)** Nomogram for survival risk prediction of 180, 365, and 545 days. The model contains eight features. **(C)** The ROC curve validation of the risk regression model at 6 time points. The different colored curves represent specific time-points. **(D,E)** KM plot of train and test dataset in which high- and low-risk groups were show as different lines. Log-rank test was used to calculate statistical significance. **(F)** Box plot of risk scores for samples of different tumor stages, tissue origin, and radiation therapy. The rank sum test and ANOVA were used to measure differences between groups.

Genes affected by mutations played an important role in the mechanism of carcinogenesis. It is meaningful to identify the markers associated with prognosis of PAAD patients from genes that are significantly differentially expressed between mutated and unmutated samples (*p-*value < 0.05). In total, we obtained 171 genes that were significantly differentially expressed by mutation-driven. We performed univariate cox regression to identify genes associated with overall survival (OS) in PAAD patients, and 53 genes were selected by controlling for *p*-value < 0.05. We further rigorously screened for these 53 genes using lasso regression and 8 genes including *SLC30A1, RBM10, PNPLA6, DSG2, CHML, DLGAP5, TTLL6*, and *PDE4DIPP5* were identified as significantly associated with patient OS (Supplementary Figure 4A). The multivariate Cox regression were performed to construct survival risk prediction model using these eight feature genes and train set, three of which, *RBM10, SLC30A1*, and *DLGAP5*, were major genes that associated with the risk of death in patients (Figure 5A). Nomograms were used to illustrate the probability of survival risk at 6, 12, and 18 months (Figure 5B). The calibration curve was also used to validate the stability of the risk prediction model (Supplementary Figure 4B). In order to identify the best predictive time point for the risk prediction model, we divided the 6–18 months period into six time periods and evaluated the prediction results using ROC curve. We found that the risk prediction result reached the maximum area under curve (AUC) value of 0.84 in the 474.5 days (Figure 5C). Further, we used multivariate Cox regression coefficients of eight genes identified by lasso regression to construct risk score models as follows: risk score = 0.65^∗^ *SLC30A1*—0.84^∗^ *RBM10*—0.27^∗^ *PNPLA6* + 0.36^∗^ *DSG2*—0.21^∗^ *CHML* + 0.54^∗^ *DLGAP5*—0.02^∗^ *TTLL6*—0.08^∗^ *PDE4DIPP5*, and calculated the risk score for each PAAD sample. The samples of train and test set were, respectively, divided into two categories (high-risk and low-risk) based on the median risk score, and we found that high-risk samples in both the training and test sets exhibited an association with poorer PAAD OS (Figures 5D,E). By combining clinical information from the PAAD sample with the risk score, we found that patients in stage II, III, and IV had a significantly higher risk score compared to stage I (Figure 5F), and found that the origin of the tumor was significantly related to the patient’s survival risk (Figure 5F), and found that patients treated with radiation have a significantly lower risk of survival than those who are not treated with radiation (Figure 5F). All these may provide support for the treatment of PAAD.

## Discussion

In this study, we have used mutational and transcriptomic data to reveal mutational features, driver genes and prognostic markers in PAAD. Statistical analysis of the mutational profile of PAAD revealed that relatively lower number of mutations occurred in non-coding regions of the genome, with most mutations occurring in coding regions affecting the structure and function of the protein. We identified 31 driver genes based on statistical test that are strongly associated with apoptosis, energy metabolism and invasion of tumor cells. Next, we constructed a co-expression network determined by driver genes, revealing the oncogene interaction mechanism and oncogenic pathways of PPAD. We further constructed a ceRNA dysregulation network using TargetScan and miRanda tools to reveal that somatic mutations on lncRNA regulate the expression of target genes at the post-transcriptional level. Using a dual screen of univariate cox regression and lasso regression, we identified eight genes that were strongly associated with the prognosis of PAAD patients despite the existence of public databases for studying the prognosis of pan-cancers (Qi et al., 2021). We also constructed a risk score model to specify the risk of survival for each patient, showing that higher risk scores have a poorer probability of survival.

Pancreatic cancer is one of the deadliest malignancies (Vincent et al., 2011). Multiple of studies have tried to reveal the pathogenesis of pancreatic cancer and discover effective treatments. For example, exploring the role of the microbiome in the occurrence, development and treatment of PAAD (Wang Y. et al., 2019), and discover the carcinogenic mechanism and possible treatments of PAAD from the perspective of genetics (Bhosale et al., 2018). The development of PAAD is influenced by multiple factors, the most critical is the occurrence of malignant mutations in the chromosomes. Malignant mutations in chromosomes, which hold the genetic material of an organism, will affect the physiological mechanisms of normal cells. Although there are numerous of research results to support the conquering of PAAD, few studies have focused on somatic mutations in the genome (Chang et al., 2014). We integrated mutagenomic and transcriptomic data to discover the oncogenic mechanisms and potential prognostic markers of PAAD, which is the rational application of multi-omics data in the era of big data. In revealing the carcinogenic mechanism, multi-omics research has more advantages than previous single-omics research.

CeRNAs are transcript that regulate each other by competing shared miRNAs. The proposal of the ceRNA competition mechanism provides a new direction for the post-transcriptional regulation of genes. Considering the important role of non-coding RNA in PAAD, we explored the impact of lncRNA mutations on the ceRNA competition network. We have identified 552 mutation-miRNA-lncRNA regulation units and constructed a ceRNA dysregulated network. Although there is not enough gene expression data (massive absence of miRNA expression data) to support our prediction results, it contributes to the exploration of the post-transcriptional regulatory mechanism of PAAD.

In conclusion, this study provided the mutational landscape of PAAD and discovered driver genes. The IL2-STAT5 signaling pathway and allograft rejection affected by mutations provide a new direction for the treatment of PAAD. Marker genes associated with patient prognosis were identified through univariate cox regression and lasso regression. We also provide a survival risk prognostic model for PAAD patients. All these findings in this study may provide theoretical guidance for the diagnosis and treatment of PAAD.

## Data Availability Statement

The datasets presented in this study can be found in online repositories. The names of the repository/repositories and accession number(s) can be found in the article/Supplementary Material.

## Author Contributions

KB, TZ, YL, BS, and XB designed the experiments and wrote the manuscript. XL, ZZ, and ZD collected and analyzed the data. ZW and YX validated the method and data. All authors read and approved the final manuscript.

## Conflict of Interest

The authors declare that the research was conducted in the absence of any commercial or financial relationships that could be construed as a potential conflict of interest.

## Publisher’s Note

All claims expressed in this article are solely those of the authors and do not necessarily represent those of their affiliated organizations, or those of the publisher, the editors and the reviewers. Any product that may be evaluated in this article, or claim that may be made by its manufacturer, is not guaranteed or endorsed by the publisher.

## Footnotes

1
https://portal.gdc.cancer.gov/

2
http://software.broadinstitute.org/gsea/msigdb

3
https://www.gencodegenes.org/

4
http://www.mirbase.org/ftp.shtml

5
http://mirtarbase.cuhk.edu.cn/

6
https://cytoscape.org/

7
http://www.targetscan.org/vert_60/

8
http://www.miranda.org/
