# Supplementary material for: Integrating Genetic and Transcriptomic Data to Reveal Pathogenesis and Prognostic Markers of Pancreatic Adenocarcinoma
Source: Front Genet. 2021 Sep 9;12:747270. doi: 10.3389/fgene.2021.747270 (PMC8458879; doi:10.3389/fgene.2021.747270)
Supplement: Supplementary file 1 [file Data_Sheet_1.docx]

**Supplementary Figures**

**
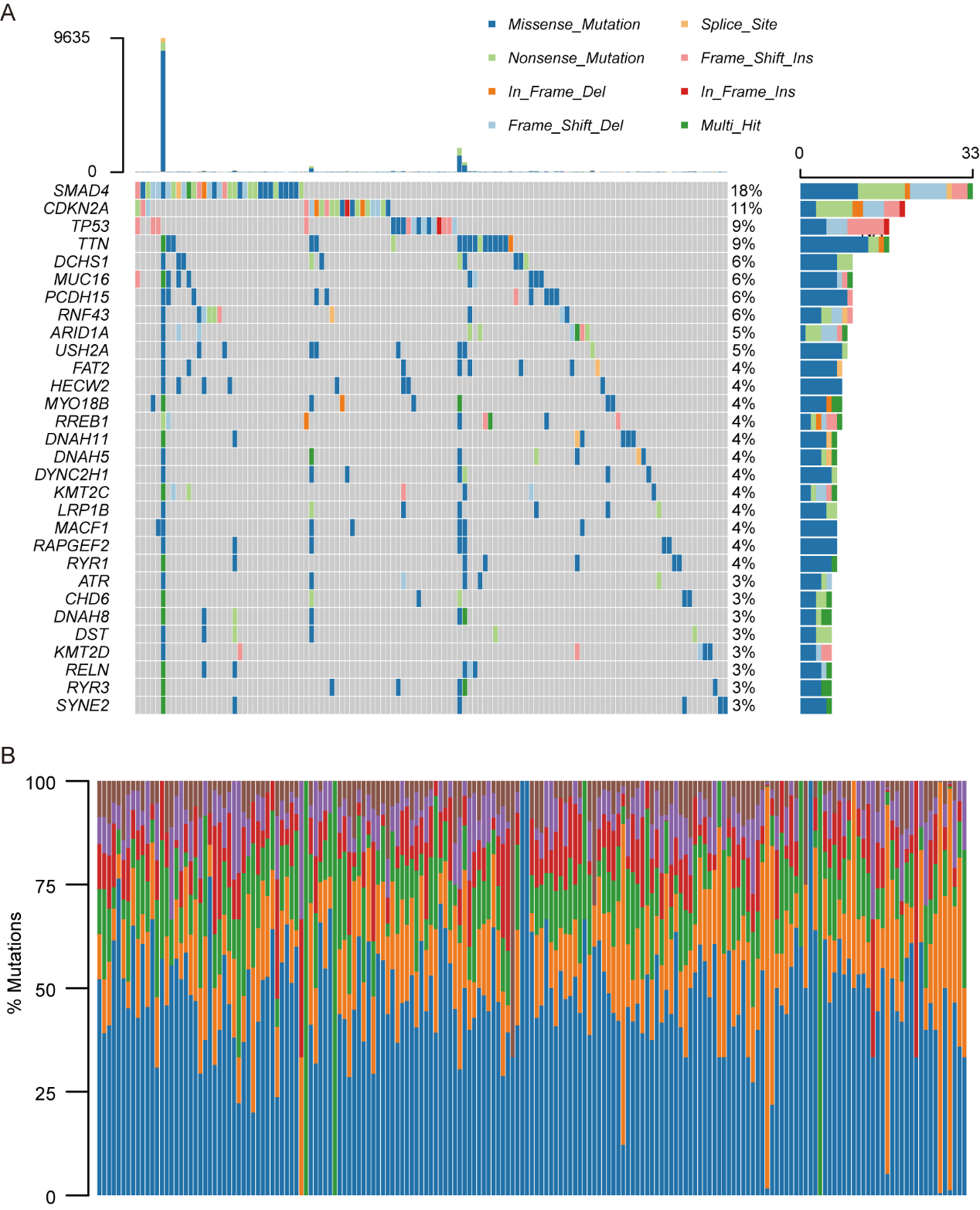
**

**Figure S1. (Related to Figure 1)** (A) The waterfall plot shows the top 30 genes in terms of mutation frequency by sample. (B) The proportion of four types of base substitutions in each mutation sample was shown by bar plot.


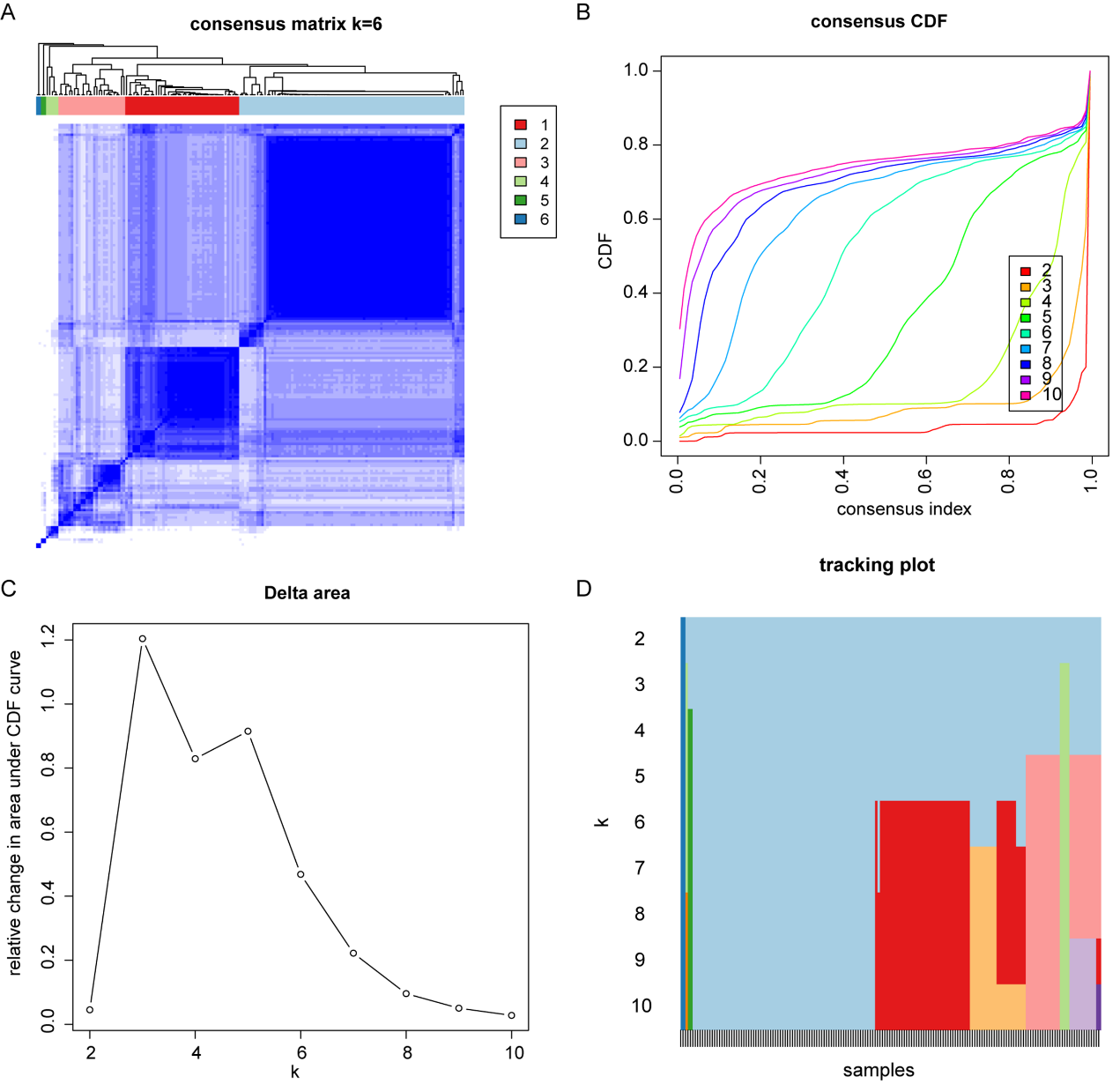


**Figure S2. (Related to Figure 2)** (A) The samples were grouped into six clusters. The consistency matrix was drawn by a heat map and the column label of the samples was added. (B) This plot described the variation curve of the CDF with the consensus index as K, which determines the number of clusters, is varied from 2 to 10. (C) The delta area score (y-axis) indicates the relative increase in cluster stability. (D) This trajectory graph reflects the change in the cluster to which each sample belongs as K moves from 2 to 10.


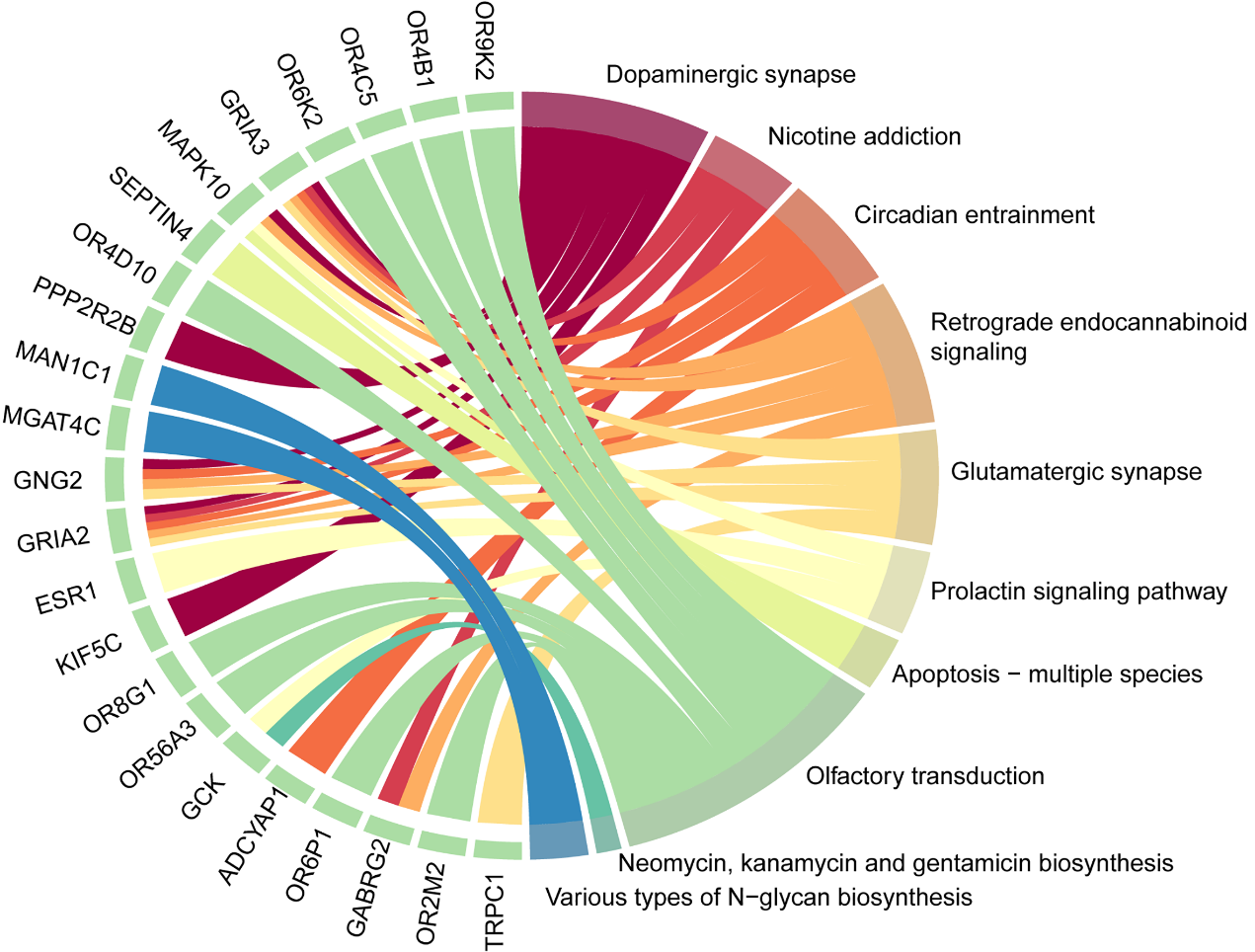


**Figure S3. (Related to Figure 3)** The top 10 KEGG pathways that are enriched by genes significantly related to driver genes. This network shows the interaction between genes and KEGG pathways.


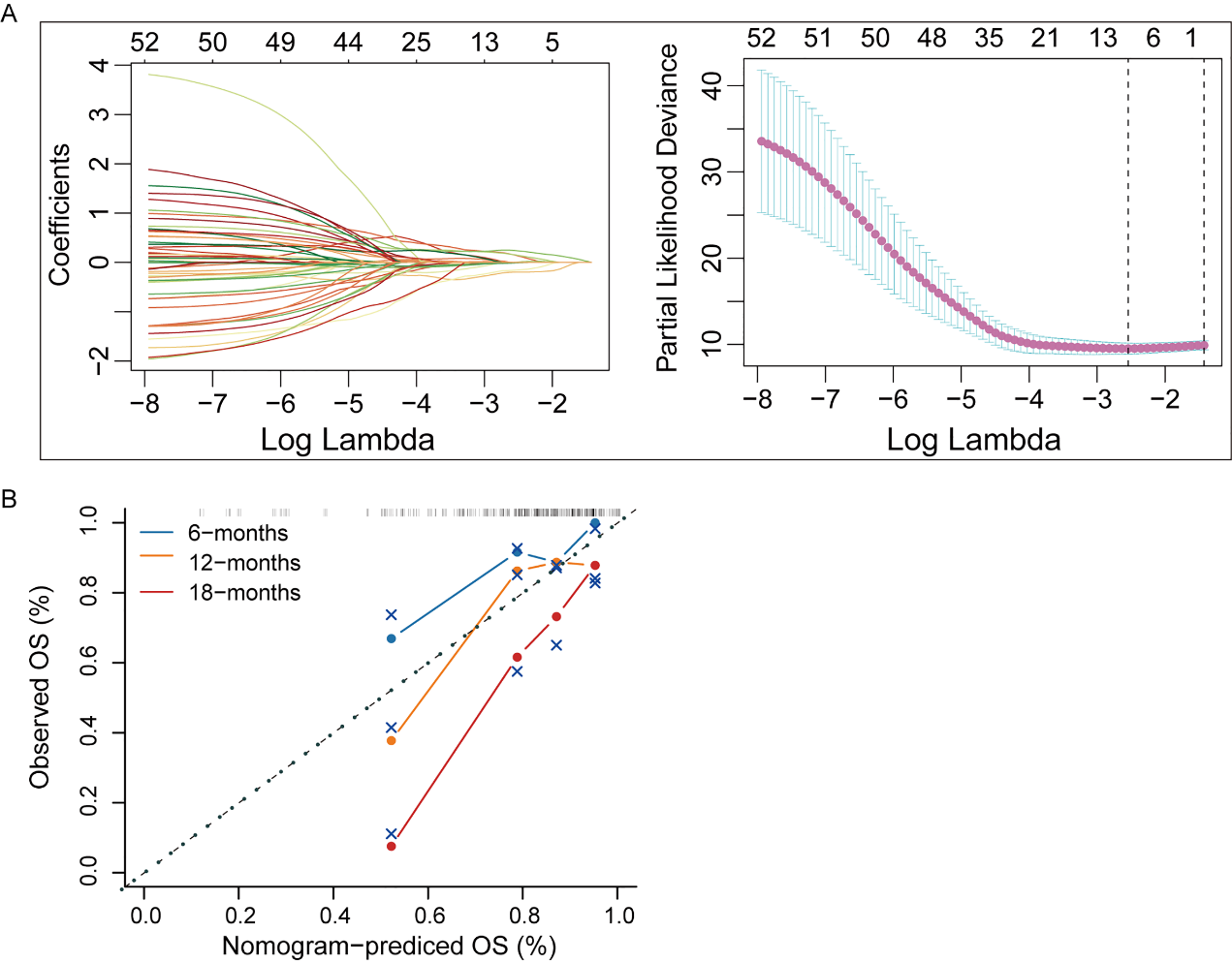


**Figure S4. (Related to Figure 5)** (A) Lasso regression model screened genes related to overall survival (OS) of patients. Variation curve of regression coefficient and β value was shown. (B) Calibration curve of nomogram.

**Supplementary Table**

**Table S1: Top 10 degree-ranked genes in the gene co-expression network**

| SYMBOL | Drgee | AverageShortestPathLength | BetweennessCentrality | ClosenessCentrality | ClusteringCoefficient | TopologicalCoefficient |
| --- | --- | --- | --- | --- | --- | --- |
| FAM133A | 192 | 1.026 | 1.000 | 0.975 | 0.000 | 0.005 |
| ZNF645 | 83 | 1.957 | 0.775 | 0.511 | 0.000 | 0.030 |
| IGKV1OR10-1 | 72 | 1.000 | 1.000 | 1.000 | 0.000 | 0.000 |
| RP11-98N22.6 | 47 | 2.472 | 0.468 | 0.404 | 0.000 | 0.022 |
| MRPL50P4 | 28 | 2.693 | 0.294 | 0.371 | 0.000 | 0.037 |
| HMGB1P18 | 17 | 1.000 | 1.000 | 1.000 | 0.000 | 0.000 |
| TGM6 | 10 | 3.785 | 0.037 | 0.264 | 0.000 | 0.700 |
| TRIM51 | 7 | 1.000 | 1.000 | 1.000 | 0.000 | 0.000 |
| Y_RNA | 7 | 2.018 | 0.615 | 0.495 | 0.000 | 0.250 |
| SPATA31A1 | 4 | 2.975 | 0.037 | 0.336 | 0.000 | 0.250 |
